# Semi-Mechanism-Based Pharmacodynamic Model for the Anti-Inflammatory Effect of Baicalein in LPS-Stimulated RAW264.7 Macrophages

**DOI:** 10.3389/fphar.2018.00793

**Published:** 2018-07-18

**Authors:** Li Xiang, Ying-Fan Hu, Jia-Si Wu, Li Wang, Wen-Ge Huang, Chen-Si Xu, Xian-Li Meng, Ping Wang

**Affiliations:** ^1^College of Pharmacy, Chengdu University of Traditional Chinese Medicine, Chengdu, China; ^2^Chengdu Pharmoko Tech LTD corp., Chengdu, China

**Keywords:** baicalein, LPS, inflammation, pharmacodynamic, TNF-α, mathematical model

## Abstract

Monitoring of the inhibition of TNF-α, IL-6, iNOS, and NO is used to effectively evaluate anti-inflammatory drugs. Baicalein was found to have good anti-inflammatory activities, but its detailed cellular pharmacodynamic events have not been expatiated by any other study. The inflammatory mediators, including TNF-α, IL-6, iNOS, and NO production in RAW264.7 macrophage induced by LPS, were measured. It was found that these data showed a sequential pattern on time and based on these points a cellular pharmacodynamic model was developed and tested. TNF-α and IL-6 were quantified by ELISA, NO was detected by Griess and iNOS expression was measured by Western blot. The pharmacodynamic model was developed using a NLME modeling program Monolix® 2016R1.^1^The results showed that baicalein quickly suppressed release of TNF-α in a concentration-dependent manner, and consequently causing the diminution of IL-6 and iNOS/NO. The pharmacodynamic model simulation successfully described the experimental data, supporting the hypothesis that IL-6 and iNOS /NO release after LPS stimulation is mediated by TNF-α rather than LPS directly. The pharmacodynamic model allowed a well understanding of the cellular pharmacodynamic mechanism of baicalein in the treatment of inflammatory diseases.

## Introduction

Inflammation, a common immune and physiological response, is caused by germs invasion, tissue injury, or cellular damage (Oh et al., 2013). In general, among the diverse immune cells which are activated by inflammatory response, macrophages play major roles in both innate and adaptive inflammation and are correlated with multiple disease such as autoimmunity, sepsis, inflammatory disorders, different liver diseases, allergy, and cancer (Tracey, 2002; Hotamisligil, 2006; Russell, 2006). Lipopolysaccharide (LPS), a primary constituent of Gram-negative bacteria cell membrane, is a significant inducer of inflammation (Heine et al., 2001; Rossol et al., 2011). Activation of macrophages by LPS results in the increase of numerous proinflammatory cytokines, such as tumor necrosis factor alpha (TNF-α), interleukin-6 (IL-6), nitric oxide (NO) (Noguchi et al., 2003; Becker et al., 2005; Kim et al., 2007; Szekanecz and Koch, 2007; Ronis et al., 2008; Jung et al., 2009). As the most proximal regulator of the cytokine cascade in inflammation, TNF-α plays an important role in antibacterial immunity (Pennica et al., 1984; Old, 1985; Creasey et al., 1991; Song et al., 2012; Caldwell et al., 2014). Interestingly, TNF-α is also one of the mediators causing iNOS overexpression in a complimentary way (ter Steege et al., 1998), which in turn, causes NO secretion (Thiemermann, 1997; Chakraborty et al., 2005). IL-6, a remarkable activator of the acute inflammatory response, is also stimulated by LPS, TNF-α, or IL-1(Nemzek et al., 2001). Produced by iNOS, NO is an important and widespread cell-signaling mediator in plenty of physiological processes (Moncada et al., 1992; Liu and Motchkiss, 1995; Tunçtan et al., 1998; Zamora et al., 2000; Sukumaran et al., 2012). Thus, these proinflammatory mediators could be worthy biomarkers on the pathogenesis, diagnosis, and prognosis of the inflammation. Studying the changes of these proinflammatory cytokines could be helpful to assess the therapeutic effect and the prognosis of various inflammatory disorders.

**Figure 1:**
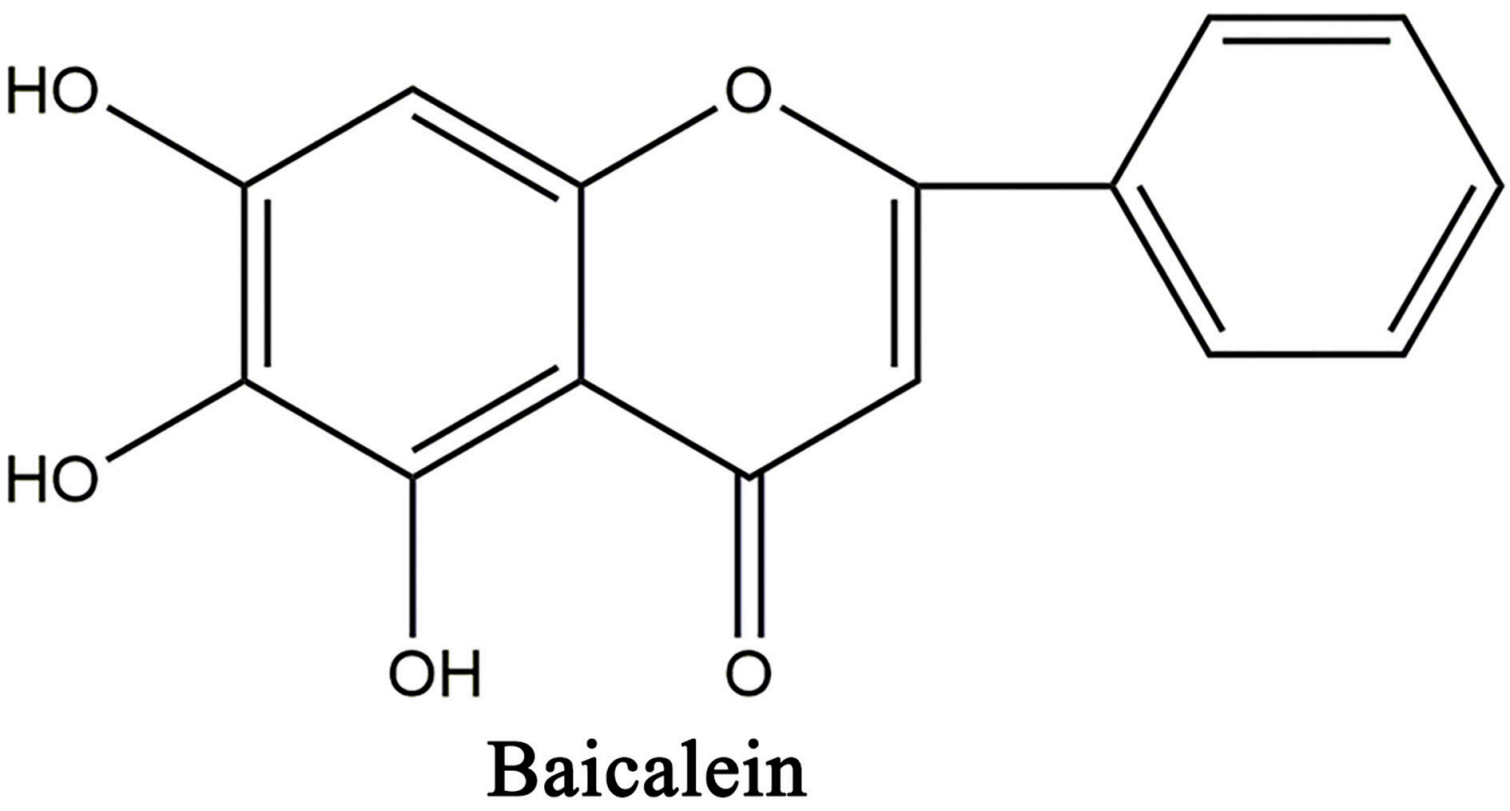
Chemical structures of baicalein.

Baicalein (5,6,7-trihydroxy-2-phenyl-4H-1-benzopyran-4-one; Figure 1) is one of the primary active flavonoid separated from the roots of *Scutellaria baicalensis Georgi*, an important Chinese medicinal herbs Huangqin. Recent investigations showed that baicalein exhibit a therapeutic effect in LPS-related multiple tissue injury including acute lung injury (Tsai et al., 2014), acute liver failure (Wu et al., 2010), myocardial dysfunction (Lee et al., 2011), and endotoxic shock in rats (Cheng et al., 2007). And baicalein was found to suppress LPS-induced TNF-α formation, iNOS mRNA expression, NO production (Wakabayashi, 1999; Kim et al., 2014), as well as IκBα phosphorylation (Wakabayashi, 1999; Chen et al., 2001; Woo et al., 2006) *in vitro* and *in vivo* (Chen et al., 2004; Woo et al., 2006; Cheng et al., 2007; Wu et al., 2010). Furthermore, it has been reported that baicalein inhibited the activation and nuclear translocation of STAT1 and STAT3 in LPS-stimulated RAW264.7 cells (Qi et al., 2013). These findings suggest that baicalein is a potential therapeutic drug for LPS-related inflammatory diseases.

Cellular pharmacodynamic, an emerging branch of traditional pharmacodynamic, recently has attracted much attention for evaluating and improving the antibiotic and anticancer drugs (Jung and Zamboni, 2001; Siebert et al., 2004; Van Bambeke et al., 2006; Barcia-Macay et al., 2008; Halder et al., 2008). In this field, cellular pharmacodynamic directly utilize the target cell as the study object to quantitatively analyze the dynamic features of drug pharmacodynamic properties in the cellular microenvironment. Subsequently, the mathematical models will be developed to describe the intracellular target ability and drug activity (Zhou et al., 2011). In this way, pharmacodynamic research could facilitate to overcome inappropriate cellular pharmacodynamic of drugs and effectively promote drug discovery by transporting from macrocosm into microcosm. However, at present, majority of the relevant studies put much emphasis on the prediction of the drugs intracellular activities, and few researches refer to the dynamic process and parameters of drugs cellular pharmacodynamic. Although, some pharmacokinetic models have been developed to explain the disposition of the drugs, the combination of pharmacodynamic properties was scarce (Hung et al., 2005; Zhang et al., 2006). It would be better to combine the drug pharmacodynamic properties with mathematical models to describe the dynamic process of intracellular drug and calculate the related pharmacodynamic parameters. More effort remains to be made to establish a pharmacodynamic model to better understand the correlation between drug efficacy and intracellular pharmacodynamic processes. (Zhang et al., 2012).

Although various anti-inflammatory activities and the related mechanism of baicalein have been reported, the detailed cellular pharmacodynamic mechanisms based on the anti-inflammatory activities of baicalein has not been proved by any other study. Therefore, in the present research, we evaluated the anti-inflammatory effect of baicalein on LPS-induced inflammation in RAW264.7 macrophage cells and develop a cellular pharmacodynamic model to fully understand the correlation between cellular pharmacodynamic properties and anti-inflammatory action of baicalein.

## Materials and methods

### Chemicals

Baicalein (purity 98.58%) was purchased from Chengdu MUST Bio-Technology Co. Ltd. (Chengdu, China). LPS from *Escherichia coli* 055: B5 and Dimethyl sulfoxide(DMSO) were obtained from Sigma-Aldrich (St. Louis, MO, USA). Fetal bovine serum (FBS) was got from Hyclone (Thermo Scientific, Waltham, MA, USA). Dulbecco's modified Eagle's medium (DMEM) was acquired from Gibco (Invitrogen, Carlsbad, CA, USA). RIPA buffer and phenylmethanesulfonyl fluoride (PMSF) were gained from Wuhan Boster Bio-Technology Co. Ltd. (Wuhan, China). 3-(4,5-dimethylthiazol-2-yl)-2,5-diphenylthiazolium bromide (MTT) was purchased from Beyotime Bio-Technology Co. Ltd. (Shanghai, China). NO Griess reagent was purchased from Beyotime (Shanghai, China). Mouse TNF-α and IL-6 ELISA kits were obtained from MULTI SCIENCES (Hangzhou, China). Rabbit monoclonal antibody against iNOS and mouse monoclonal antibody against Glyceraldehyde 3-phosphate dehydrogenase (GAPDH) were acquired from Abcam (Abcam Limited, Cambridge, United Kingdom). Peroxidase-conjugated affinipure goat anti-rabbit IgG and peroxidase-conjugated affinipure goat anti- anti-mouse IgG were obtained from Zen BioScience Co. Ltd (Chengdu, China). All other reagents were analytical grade.

### Cell culture

Murine macrophage RAW 264.7 cells were acquired from the Center of Cellular Resources, Chinese Academy of Sciences (Shanghai, China). All cells were cultured in cell culture medium (DMEM supplemented with 10% FBS and 1% penicillin–streptomycin) and incubated in a humidified incubator with a 5% CO_2_ atmosphere at 37°C.

### MTT assay

Cell viability was evaluated by MTT assay. In brief, 1.5 × 10^5^ RAW264.7 cells were inoculated into 96-well plates and incubated at 37°C with 5% CO_2_ overnight. Blank wells (cell culture medium) was reserved in advance. Different concentrations of baicalein (0, 10, 20, and 40 μM) mixed with LPS (1 μg/mL) were added to the cells followed by incubation for 1, 2, 4, 8, 12, and 24 h, respectively. And then, the cells in each well were added MTT solutions and cultured for another 4 h. After the cells dissolving in dimethyl sulfoxide (DMSO), the optical density (OD) was read at 490 nm using microplate reader (Thermo, MμLtiskan MK3, USA). OD values of the cell were defined as the OD values in test wells compared to the blank wells. Besides, the cell numbers in a random view in each well were counted under the microscope. Reported data are the mean of sextuplicate analyses.

### Measurement of the production of TNF-α, IL-6 and NO

RAW 264.7 cells with 1 × 10^6^ cells/mL, at logarithmic phase, were inoculated into 24-well plates overnight, and pretreated with three concentrations of baicalein (10, 20, and 40 μM) for 0.5 h, followed by stimulation with 1 μg/mL LPS for additional 1, 2, 4, 8, 12, and 24 h, respectively. After stimulation, cell culture media were collected and centrifuged at 300 g for 10 min at 4°C. The supernatants were sampled for TNF-α, IL-6 and NO assays. Concentrations of TNF-α and IL-6 were quantitated using a Mouse TNF-α and IL-6 ELISA kits following manufacturer protocols, respectively. The intra and inter assay variability for the assays of TNF-α and IL-6 were 2.3~3.0 and 2.1~5.3, respectively. NO was quantitated by detecting its stable oxidative metabolite, nitrite. Briefly, 50 μL culture media was blended with Griess reagent (Beyotime) of the same volume and incubated for 30 min at 37°C. Absorbance was read at 540 nm by the microplate reader. The nitrite concentration was calculated by the calibration curve using sodium nitrite standard solutions (Lin et al., 2013).

### Western blotting

RAW 264.7 cells with 1 × 10^6^ cells/mL, at logarithmic phase, were inoculated into 6-well plates overnight, and pretreated with three concentrations of baicalein (40, 20, and 10 μM) for 0.5 h, followed by stimulation with 1 μg/mL LPS. After another 0, 1, 2, 4, 8, 12, and 24 h incubation, respectively, the cells were washed with cold phosphate-buffered saline (PBS) and then lysed in 125 μL RIPA buffer added with PMSF and cocktail of protease and phosphatase inhibitors (Pierce, Rockford, IL, USA). Cell samples were collected into 0.5 mL centrifuge tubes and separated by centrifugation at 1 × 10^4^ rpm for 10 min at 4°C. The supernatants were sampled and stored at −80°C until analysis.

The contents of protein were detected by the bicinchoninic acid method using BCA protein assay kit (Pierce Chemical). Aliquots of total cell lysates were blended in loading buffer and boiled for 5 min for the protein denaturation. Equivalent amounts of denatured protein were separated by 10% SDS-PAGE electrophoresis, subsequently transferred to PVDF membrane, and then blocked with 5% bovine serum albumin (BSA) under room temperature for 1 h. After blocking, membranes were incubated with the primary monoclonal antibody against iNOS and GAPDH (both at 1:1000 dilution) respectively, overnight at 4°C. After being washed, the PVDF membranes were subsequently incubated with HRP-conjugated secondary antibody (goat anti-rabbit or goat anti-mouse antibodies, 1:5000) under room temperature for 1 h. After being marked by an enhanced chemiluminescence kit (MULTI SCIENCES (Hangzhou, China), the chemiluminescent signal was detected by Bio-Rad ChemiDoc™ XRS+ System (Bio-Rad, USA). The intensity of each signal was quantified by a computer image analysis system (Quantity One, Bio-Rad). The expression levels of iNOS at different time points were standardized by the iNOS level at 0 h point (control), which was described by the following equation.

$$\begin{array}{l} \text{Relative iNO Sratio}=\frac{\text{iNOS}/\text{GAPDH}}{\text{iNO}{\text{S}}_{0}/\text{GAPD}{\text{H}}_{0}} \end{array}$$

### Pharmacodynamic modeling

**Figure 2:**
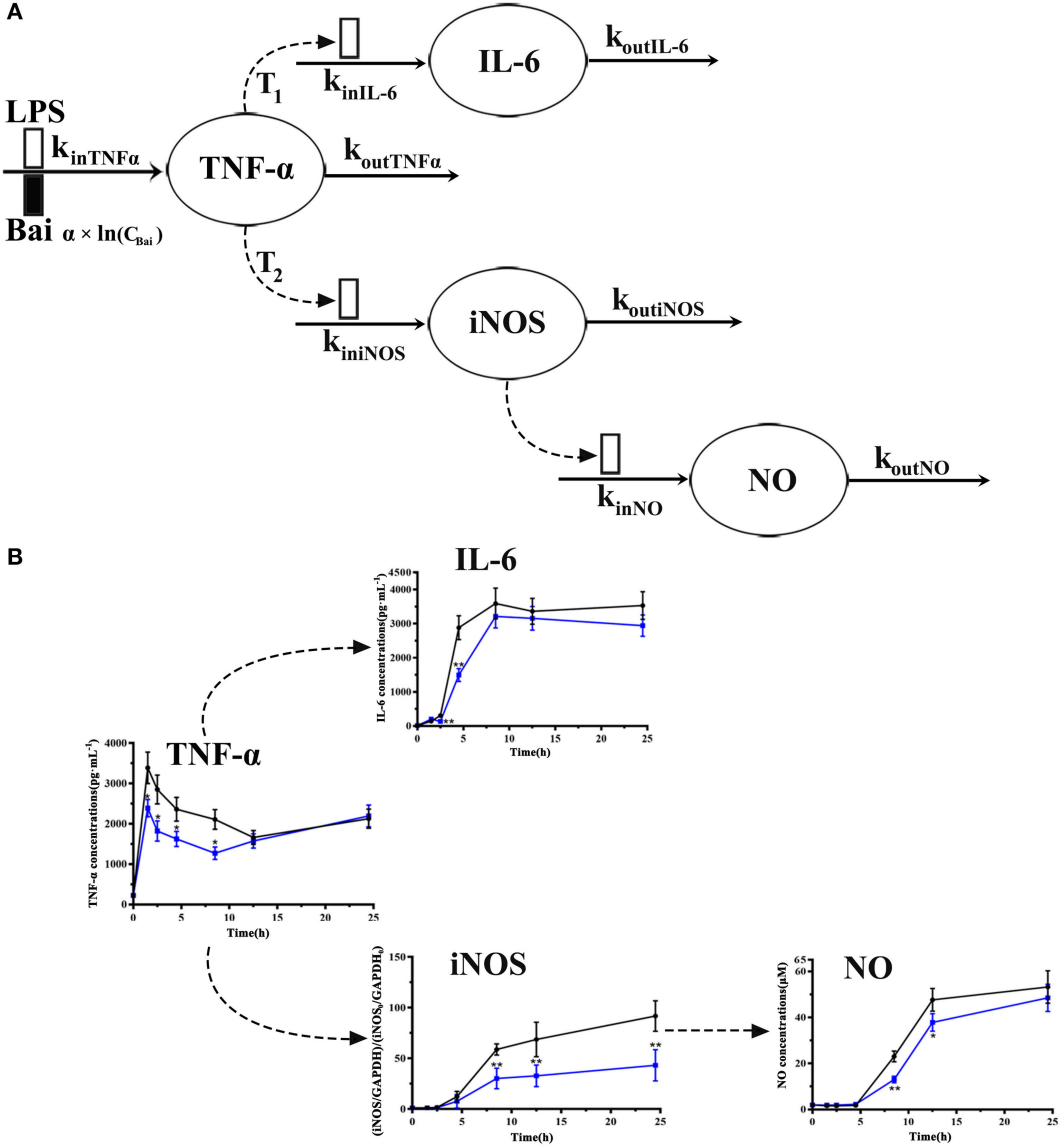
Model schematic of the pharmacodynamic model for the effects of LPS and baicalein on TNF-α, IL-6, and NO production and iNOS expression dynamics **(A)**. Boxes reflects stimulation (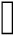) and inhibition of production rate (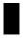) of TNF-α. Concentrations of TNF-α, IL-6, iNOS and NO level on each time point **(B)**. The black line represents the model group stimulated by LPS, and the blue line represents dada of baicalein (40 μM) inhibiting stimulation of LPS.

The schematic diagram of the integrated pharmacodynamic model that described the dynamic changes of the TNF-α, IL-6, NO in culture media supernatant and iNOS expression in cells is shown in Figure 2. Pharmacodynamic analysis was implemented with a non-liner mixed effects modeling (NLME) program, Monolix® 2016R1 software (Lixoft, France). The first-order conditional estimation with the extended least-squares (FOCE-ELS) method was carried out to estimate the pharmacodynamic parameters and their variability. Structural pharmacodynamic model selection and diverse weighting strategies (additive, power, multiplicative) were all based on goodness-of-fit plots, Akaike's information criteria (AIC), comparison of the observed and individual predicted concentrations, and the coefficient of variation (CV) in parameter estimations.

The dynamic changes of TNF-α concentrations were modeled by the indirect response model with LPS stimulation and baicalein reducing its release (Dayneka et al., 1993). The model is shown in the following equations:

(1) $$\begin{array}{rcl} \frac{dTNF\alpha }{dt} & = & k_{inTNF\alpha }\times \left(1-f\left(Bai\right)\right)-{\text{k}}_{\text{outTNFα}}\times \text{ TNFα} \end{array}$$

(2) $$\begin{array}{rcl} f\left(Bai\right) & = & \alpha \;\times \;\ln\left(C_{Bai}\right) \end{array}$$

(3) $$\begin{array}{rcl} {TNF\alpha }_{0} & = & Control\left(TNF\alpha \right) \end{array}$$

where k_inTNFα_ represents the zero-order rate constant of the TNF-α production, it reflects the effect of LPS stimulation on induction of TNF-α. The inhibition of baicalein on TNF-α production was described by Equation (2) (f(Bai)), which was assumed that the inhibition effect of baicalein has a logarithmic linear correlation with the TNF-α concentration. TNFα represents the concentration in culture media supernatant, and TNFα_0_ is the TNF-α production at the point of 0 h (control), which was shown in Equation (3). This model was proposed by *Wyska* to show the inhibitory effect of methylxanthine derivatives on TNF-α production in LPS-stimulated mice (Wyska, 2010).

Macrophages also secrete IL-6, a remarkable activator of the acute inflammatory response. It has been reported that IL-6 production is induced by TNF-α as well as LPS (Nemzek et al., 2001). It is also found that the production of IL-6 was mediated by TNF-α (Yao et al., 1997). Based on these reports and our observed data sequential pattern, an indirect response model was carried out in this part and described by the following equations:

(4) $$\begin{array}{rcl} \frac{\text{dIL}-6}{\text{dt}} & = & {\text{k}}_{\text{inIL}-6}\times \text{ TNFα}\left(t-\tau_{1}\right)-{\text{k}}_{\text{outIL}-6}\times \text{ IL}-6 \end{array}$$

(5) $$\begin{array}{rcl} \text{IL}-6_{0} & = & \text{Control}\left(\text{IL}-6\right) \end{array}$$

where TNF-α represents the TNF-α concentration, k_inIL−6_ is an intercompartmental rate constant describing the secretion of IL-6 from TNF-α, k_outIL−6_ represents the first-order elimination rate constant of IL-6. The τ_1_ is the lag time in the production of IL-6 from TNF-α. IL-6_0_ is the IL-6 production at the point of 0 h (control), which was shown in equation (5).

Previous researches have shown that TNF-α is one of the mediators resulting in the iNOS overexpression in a complimentary way (ter Steege et al., 1998), which in turn, causes NO secretion (Chakraborty et al., 2005). Besides, TNF-α induced the overexpression of iNOS by activating nuclear factor-kappa B (NF-κB) (Thiemermann, 1997). Based on these reports and our observed data of TNF-α and iNOS sequential cascade, the dynamic changes of iNOS expression were modeled with TNF-α inducing the iNOS secretion. The model is described by the following equations:

(6) $$\begin{array}{r} \frac{\text{diNOS}}{\text{dt}}={\text{k}}_{\text{iniNOS}}\times \text{ TNF}\alpha \left(t-\tau_{2}\right)-{\text{k}}_{\text{outiNOS}}\times \text{ iNOS} \end{array}$$

Where TNF-α represents the TNF-α concentration, k_iniNOS_ is the intercompartmental rate constant representing the production of iNOS from TNF-α. The τ_2_ is the lag time in the production of iNOS from TNF-α. K_outiNOS_ is the first-order elimination rate constant of iNOS.

The dynamic change of NO production was modeled by an indirect response model with linear systemic elimination. The equations were shown below:

(7) $$\begin{array}{r} \frac{\text{dNO}}{\text{dt}}={\text{k}}_{\text{inNO}}\times \;{\text{iNOS}}^{\text{delta}}-{\text{k}}_{\text{outNO}}\times \text{ NO} \end{array}$$

Where iNOS represents the expression of iNOS. k_inNO_ is the intercompartmental rate constant describing the production of NO from iNOS, k_outNO_ is the first-order elimination rate constant of NO. The delta is the exponential term of NO from iNOS expression, which expresses an amplifying effect of iNOS to NO production. A similar model was applied by Sukumaran et al. (2012).

Since the levels of TNF-α, IL-6, iNOS, and NO showed no difference compared with the levels in the model after 24.5 h treatment of baicalein, only the data of TNF-α, IL-6, iNOS, and NO production after 1.5, 2.5, 4.5, 8.5, and 12.5 h (without 24.5 h) treatment of baicalein were used in this model. To check the stability of the final pharmacodynamic model, the model was assessed by Monolix® 2016R1 software (Lixoft, France).

### Statistical analysis

All data were graphically performed by GraphPad Prism 7.0 (GraphPad, Avenida, CA, USA) and statistically analyzed using IBM SPSS 23.0 software. Data were presented as the mean ± standard deviation (SD) of individual values from three independent experiments. One-way analysis of variance (ANOVA) followed by a Dunnett post hoc test was applied for significance analysis. The differences were adjudged statistically significant when *P* < *0.05* and very significant when *P* < *0.01*.

## Results

### Analysis of the data sequential pattern of TNF-α, IL-6, iNOS, and NO

To explore cellular pharmacokinetic mechanisms underlying the anti-inflammatory action of baicalein, we firstly analyze the data changing pattern on each time point of LPS-induced inflammatory mediators. As shown in Figure 2B, firstly, the stimulus of LPS caused immediate release of TNF-α, which rapidly increased to peak at 1 h and gradually declined until 12 h. And then TNF-α had a slow rise during 12–24 h after LPS stimulated. IL-6 increased slowly between 1 and 2 h after stimulating, but significantly reached a peak at 4 h. Subsequently, after LPS stimulated, the concentration of IL-6 peaked at 8.5 h and barely changed until the last observing time. The iNOS expression showed a similar change comparing with IL-6 except a delayed increase. As we known, the change of NO release correlated with the iNOS contents. Furthermore, the present study also showed that different doses of baicalein, especially 40 μM, could reduce the elevated level of TNF-α, IL-6, iNOS and NO (*P* < *0.05* and *P* < *0.01*). Besides, cell viability and cell counts analysis showed that baicalein did not affect the reduced cell viability (OD value of MTT) and cell counts induced by LPS (shown in Supplementary Material).

### Model simulation

**Figure 3:**
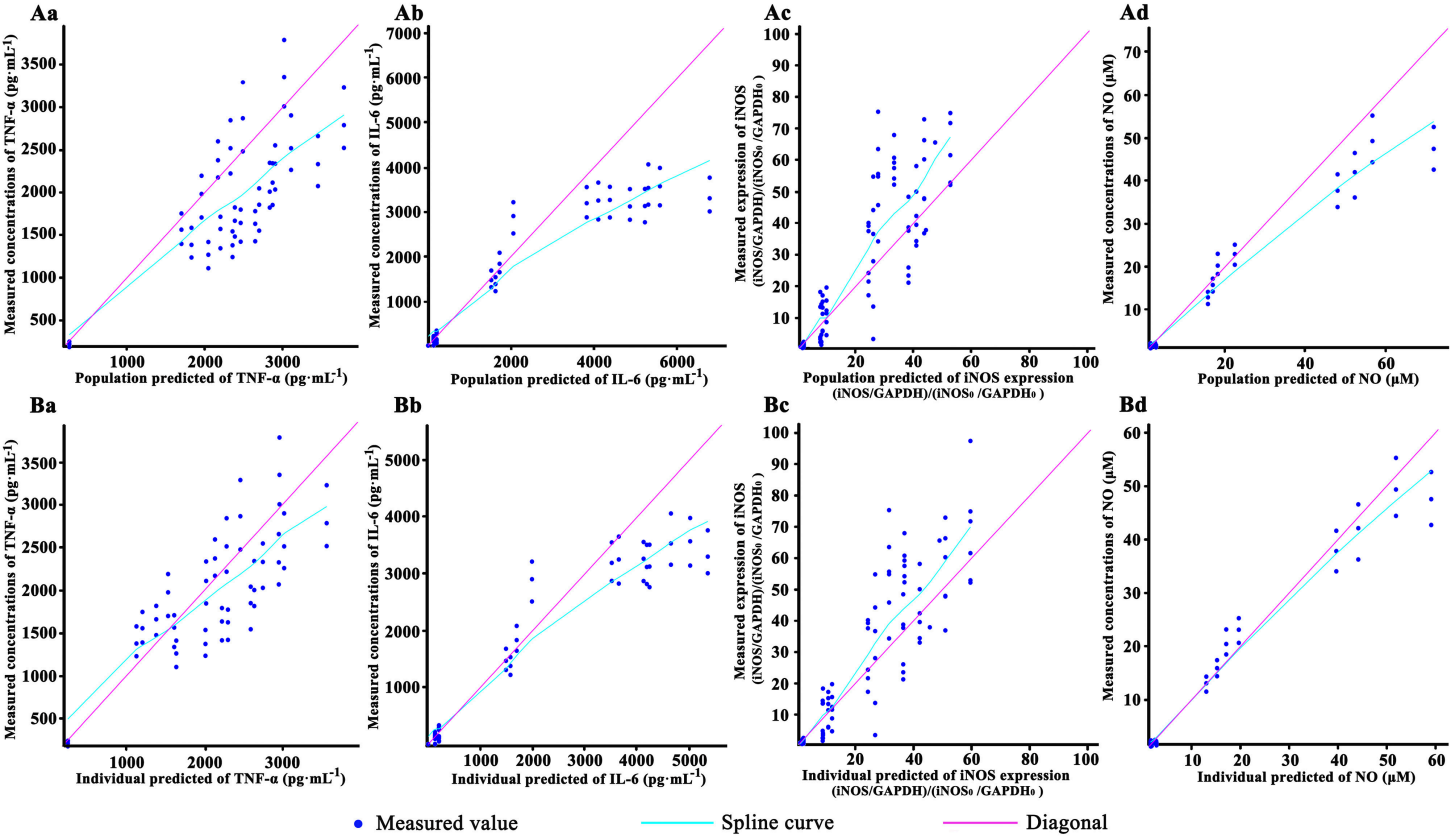
The goodness-of-fit plots of the pharmacodynamic model: Relationship between observed and predicted population values of TNF-α **(Aa)**, IL-6**(Ab)**, iNOS **(Ac)** and NO **(Ad)**; Relationship between observed and predicted individual values of TNF-α **(Ba)**, IL-6**(Bb)**, iNOS **(Bc)**, and NO **(Bd)**. Cells were incubated with 40, 20, 10, and 0 (control) μM baicalein for 1.5, 2.5, 4.5, 8.5, 12.5, and 24.5 h. Except the control, the RAW264.7 macrophages cells were stimulated with LPS 0.5 h after incubating with baicalein. TNF-α and IL-6 concentration were quantified by ELISA, NO was detected by Griess and iNOS expression was measured by Western blot. For all of the sub-graphs, the pale rose solid lines represent the diagonal (x = y line), and the blue solid lines represent the spline curve. For experimental data points, except iNOS (*n* = 6), *n* = 3.

The goodness-of-fit plots for the pharmacodynamic model are shown in Figure 3. In this figure, the dots of observed values respectively vs. the population predicted value (PRED) and individual predicted value (IPRE). The trend lines of both population and individual predicted values of TNF-α, IL-6 were well close to x = y. Besides, the trend lines of individual predicted values of iNOS and NO also close to x = y. The observed values of the TNF-α, IL-6, iNOS, and NO were well correlated with the PRED and IPRE.

The observed data of iNOS expression and NO level maintained a proper balance after 12.5 h treatment of baicalein, k_outiNOS_, and k_outNO_ were fixed to 0 to reduce the model volatility. In general, the maximum coefficient of variation (CV%) of the estimate parameters was 37%, −2xlog-likelihood was 2818.03, Akaike Information Criteria (AIC) was2866.03, and Bayesian Information Criteria (BIC) was 2873.29, which mean the model shown a good estimation with appropriate precision. This illustrated that the model well described the data pattern of the effects of baicalein on TNF-α and the next cascaded regulatory of IL-6, iNOS, and NO.

The estimates of pharmacodynamic parameters are listed in Table 1. In this model, one of the important parameter α was 0.0832 (15 CV%), representing the logarithm-linear coefficient of TNF-α inhibited by baicalein. The low value of α showed that TNF-α inhibition may just increase about 8.32% for each degree rise in baicalein concentration, which suggested that baicalein may have a weak inhibitory effect on TNF-α.The zero-order rate constant of the TNF-α production (k_inTNFα_) was 3.74 × 10^3^ h^−1^(6 CV%), which suggested that TNF-α production is very fast to respond to LPS stimulation.

**Table 1:** Estimates of parameters for Pharmacodynamics models.

**Parameter**	**Definition**	**Estimate**	**CV%**
*a*	Linear coefficient of TNF-α inhibition induced by logarithm concentration of baicalein	0.0832	15
*k_*inTNFα*_ (h^−1^)*	Production constant of TNF-α induced by LPS	3.74 × 10^3^	6
*k_*outTNFα*_ (h^−1^)*	Elimination constant of TNF-α	0.0463	32
*k_*inIL*−6_ (h^−1^)*	Production constant of IL-6 induced by TNF-α	0.353	9
*k_*outIL*−6_ (h^−1^)*	Elimination constant of IL-6	0.143	23
*τ_1_*	Lag time in the production of IL-6 from TNF-α	1.38	11
*k_*iniNOS*_(h^−1^)*	Production constant of iNOS from TNF-α	0.00169	9
*τ_2_*	Lag time in the production of iNOS from TNF-α	1.41	13
*k_*inNO*_(h^−1^)*	Production constant of NO from iNOS	0.0605	37
*delta*	Exponential term of NO from iNOS	1.35	9

### Pharmacodynamics model

**Figure 4:**
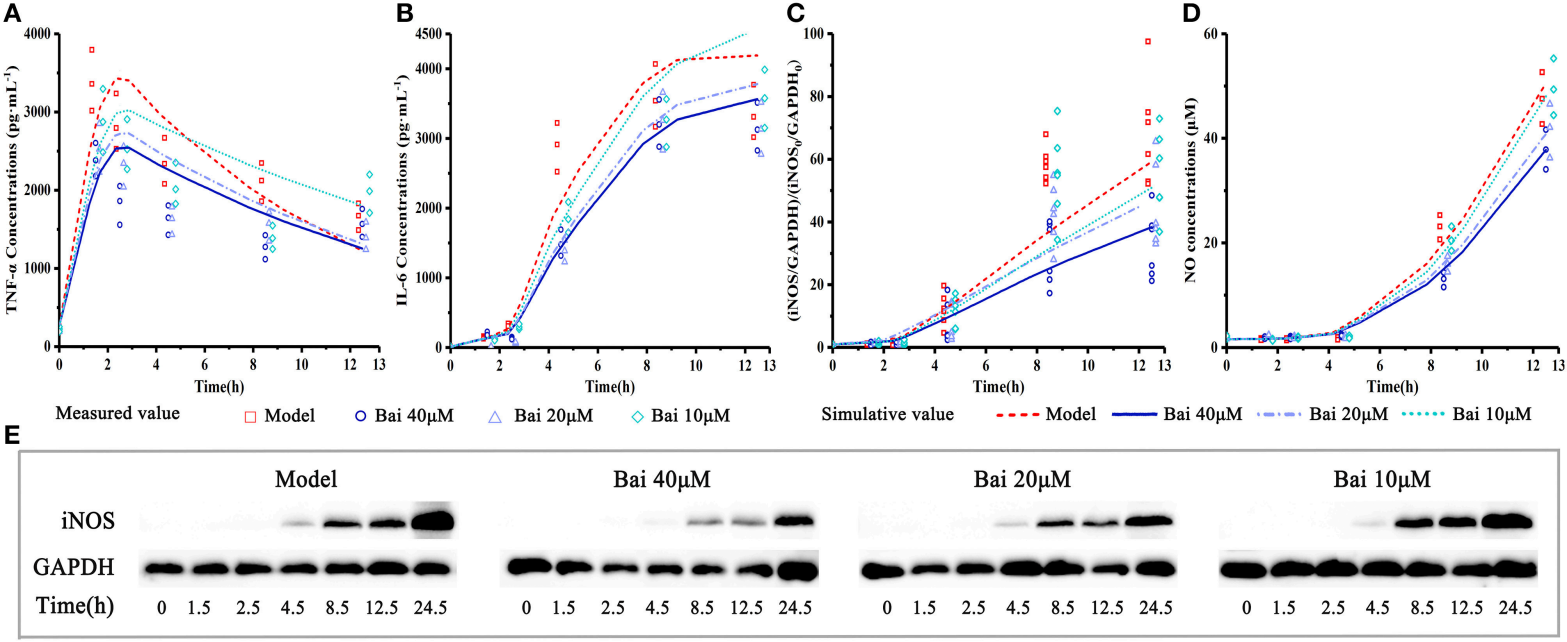
Experiment measured and model-simulated TNF-α, IL-6 and NO concentration in cell culture media and iNOS expression in cell. Cells were incubated with 40, 20, 10, and 0 (control) μM baicalein for 1.5, 2.5, 4.5, 8.5, 12.5, and 24.5 h. Except the control, the RAW264.7 macrophages cells were stimulated with LPS 0.5 h after incubating with baicalein. TNF-α **(A)** and IL-6 **(B)** concentration were quantified by ELISA, iNOS expression **(C,E)** was measured by Western blot and NO **(D)** was detected by Griess. The pharmacodynamic model of the TNF-α production network accounts for experimental data. Measured values and model fittings were shown in symbols and lines, respectively. For experimental data points, except iNOS (*n* = 6), *n* = 3.

The pharmacodynamics model reasonably described the pharmacodynamic profiles of these data. The observed and model-simulated TNF-α, IL-6, and NO concentration and iNOS expression are graphically presented in Figure 4. In our pharmacodynamic model, the anti-inflammatory property of baicalein was log-linearly related to TNF-α in a concentration-dependent manner, that is, the elevated TNF-α just be reduced about 10% for each degree rise in baicalein concentration, and the elevated IL-6, iNOS and NO production may be reduced about 10% for each degree rise in TNF-α production as well. The results showed that baicalein quickly suppressed release of TNF-α in a concentration-dependent manner, and consequently causing the diminution of IL-6 and iNOS/NO. The pharmacodynamic model simulation successfully described the experimental data, supporting the hypothesis that IL-6 and iNOS /NO release after LPS stimulation is mediated by TNF-α rather than LPS directly.

## Discussion

Inflammation has been generally considered as an immune response to multiple pathological conditions, which exists in various diseases (Bradford et al., 2015). Proinflammatory cytokines such as TNF-α, IL-6, and NO are important biomarkers in inflammation and monitoring these biomarkers' release is commonly used to evaluate anti-inflammatory drugs (Song et al., 2012; Sukumaran et al., 2012; Raposo et al., 2013; Lu et al., 2015). In recent years, baicalein was found to have anti-inflammatory activities (Wakabayashi, 1999; Chen et al., 2001, 2004; Woo et al., 2006; Cheng et al., 2007; Wu et al., 2010). But its detailed pharmacodynamic mechanisms especially underlying the anti-inflammatory action remains unclear.

The results showed that the stimulus of LPS caused immediate release of TNF-α in RAW264.7 macrophage, which rapidly increased to peak at 1 h after LPS stimulus. This result is consistent with the researches of Song R. et al. which showed that TNF-α in serum of beagles peaked at 1 h and rapidly decreased by 6 h after administration of LPS (Song et al., 2012). Previous research has also shown that the time required to trigger the TNF-α secretion from the monocytes activated by LPS is only 5 ± 15 min (Gallay et al., 1993). These results indicated that TNF-α is the earlier inflammatory cytokine that activated by LPS in macrophage compared with IL-6, iNOS, and NO. Moreover, some previous studies have shown that TNF-α is one of the mediators causing iNOS overexpression, which in turn, causes NO release. Besides, TNF-α activated NF-κB, and brought the overexpression of iNOS (Kubo et al., 1994; Asai et al., 1996; Chen et al., 1996). Similar to iNOS, it is also found that the production of IL-6 was mediated by TNF-α. Yao YM et al. found that the activation and the release of IL-6, at least in part, be mediated via TNF-dependent mechanisms (Yao et al., 1997).

Various lines of evidence have shown that mathematical models studying the interaction between TNF-α and NO in LPS-induced inflammation have enhanced the interpretation of the data. Veszelovszky et al. developed a PK-PD model to describe the development of TNF-α concentration induced by FAA, the increased iNOS production in answer to TNF-α (Veszelovsky et al., 1995). Chakraborty et al. modeled the effect of IL-10 with prednisolone on concentration of NO in mouse after LPS administration with noncompetitive interaction models. They described the production of TNF-α and IFN-γ respond to LPS administration, and the secretion of NO was a cascading result of the TNF-α and IFN-γ concentrations in plasma (Chakraborty et al., 2005).

Based on these researches and our results about the sequential pattern of these cytokines release, we established the pharmacodynamic model in two separate ways: (a) TNF-α is the early and crucial cytokine responding to stimulation of LPS; (b) and then secretions of IL-6 and iNOS are mediated by TNF-α rather than by LPS directly. And the data showed that production of TNF-α is very fast after LPS stimulation, a zero-order kinetic is used to describe the process of producing TNF-α.

In our pharmacodynamic model, the anti-inflammatory property of baicalein was logarithmly related to TNF-α. The quantitative relationship of TNF-α with IL-6, iNOS, and NO could be described by our model well. This may provide an effective method from a unique perspective to understand the pharmacodynamic relevance and mechanism of baicalein efficacy. Although, the simulative result of iNOS expression seemed not enough accurate, it still showed the trend of prediction. We speculated that the quantitative method, which used three continuing division to express iNOS mass [(iNOS/GAPDH)/(iNOS_0_/GAPDH_0_)], would make some deviation signal to the model parameters estimation.

The pharmacodynamic model could provide a more intuitive viewpoint on where and how the baicalein is targeting and its potency in a quantitative way. Meanwhile, this model may be useful for arranging baicalein's dose regimen. The pharmacodynamic model concentrated on the pro-inflammatory factors and directly connected these cytokines response to anti-inflammation, which could provide a deep understanding of the pharmacological activity of drugs. In the present study, the observed values at different dosages of baicalein were well predicted by the model.

## Conclusion

In conclusion, the pharmacodynamic model simulation successfully described the experimental data, supporting the data's cascading pattern after LPS stimulation in RAW264.7 macrophages. The reason for the delay of IL-6 and iNOS/NO production after LPS stimulation may be mediated by TNF-α rather than by LPS. Baicalein showed a weak anti-inflammatory property. It inhibited the release of TNF-α in LPS-stimulated RAW264.7 in a concentration-dependent manner, and then sequentially decrease the production of IL-6, iNOS, and NO. The pharmacodynamic model allowed the better understanding of the pharmacological potential and cellular pharmacodynamic mechanisms of baicalein in the treatment of inflammatory disorders.

## Author contributions

LX, PW, and X-LM conceived and designed the experiments; LX conducted the experiments and wrote the manuscript; LX, Y-FH, J-SW, LW, and W-GH performed the experiments; PW and C-SX analyzed the data generated from the experiments, conducted statistical analysis; PW and X-LM participated in the critical analysis of the data and revisions of the manuscript. All authors reviewed and approved the final manuscript.

### Conflict of interest statement

The authors declare that the research was conducted in the absence of any commercial or financial relationships that could be construed as a potential conflict of interest. The reviewer SU and handling Editor declared their shared affiliation.
