# Supplementary material for: Semi-Mechanism-Based Pharmacodynamic Model for the Anti-Inflammatory Effect of Baicalein in LPS-Stimulated RAW264.7 Macrophages
Source: Front Pharmacol. 2018 Jul 18;9:793. doi: 10.3389/fphar.2018.00793 (PMC6058255; doi:10.3389/fphar.2018.00793)
Supplement: Supplementary file 1 [file Presentation_1.PDF]

Supplementary materials for

Semi-mechanism-based Pharmacodynamic Model for the Anti-inflammatory Effect of Baicalein in LPS-stimulated RAW264.7 Macrophages

Li Xiang [1], Ying-Fan Hu [1], Jia-Si Wu [1], Li Wang [1], Wen-Ge Huang [1], Chen-Si Xu [2], Xian-Li Meng [1] and Ping Wang [1*]*

[1] College of Pharmacy, Chengdu University of Traditional Chinese Medicine, Chengdu 611137, China, [2] Chengdu Pharmoko Tech LTD corp, Chengdu 610041, China

This file includes:

Supplementary Text
Fig. S1. Effect of baicalein on the experiment measured and model-simulated cell viability and cell counts in LPS induced RAW264.7 macrophages.
Fig. S2. The goodness-of-fit plots of the pharmacodynamic model: Relationship between observed and predicted population values of cell viability (Aa) and cell counts (Ab); Relationship between observed and predicted individual values of cell viability (Ba) and cell counts (Bb).
Table S1 Estimates of parameters of cell viability and cell counts for Pharmacodynamics models.
Fig. S3. Effects of baicalein on IL-6 concentrations after an early and delay stimulation of LPS.
Fig. S4. Effects of baicalein on NO concentrations after an early and delay stimulation of LPS.

Supplementary Text

The cell viability (OD_{490nm} value of MTT) and cell counts analysis showed that both of them could be reduced by LPS, but could not be improved by baicalein. Based on the action of LPS on the cell viability and cell counts, the dynamic changes of the cell viability and cell counts were modeled using an indirect response model. The model is described by the following equations. The natural growth of the cell in the control was described by equation (1), the growth of the cell co-incubated with LPS and baicalein was described by equation (2), and cell counts was described by equation (3).

$$Cell\ viability = \alpha \times e^{\beta t} \qquad t \to \infty \qquad (1)$$

$$Cell\ viability_{\ with\ LPS} = \alpha \times e^{\beta \gamma t} \qquad t \to \infty \qquad (2)$$

$$Cell\ counts = d \times e^{f \cdot Cell\ viability} \qquad (3)$$

where α is the exponential growth rate constant of the cell viability, β represents the amplificatory coefficient of the exponential equation of cell viability, γ is the constant of inhibitory effect of LPS on the cell, d is the exponential growth rate constant of the cell counts, and f represents the amplificatory coefficient of the exponential equation of cell counts. In this model, cell growth is

*** Correspondence:**

Xian-Li Meng: xlm999@cdutcm.edu.cn; *Ping Wang:* viviansector@aliyun.com

exponential and cell counts has a logarithmic linear correlation with cell viability.

The measured and model-simulated cell viability and cell counts in LPS are shown in **Figure S1**. In the model simulation of the cell viability and cell counts, the inhibitory effects of LPS on cell viability and cell counts could be described by the model, which provides an effective approach to understand the action of LPS on cell viability and cell counts. The cell viability and cell counts could be reduced by LPS, however both of them could not be improved by baicalein.

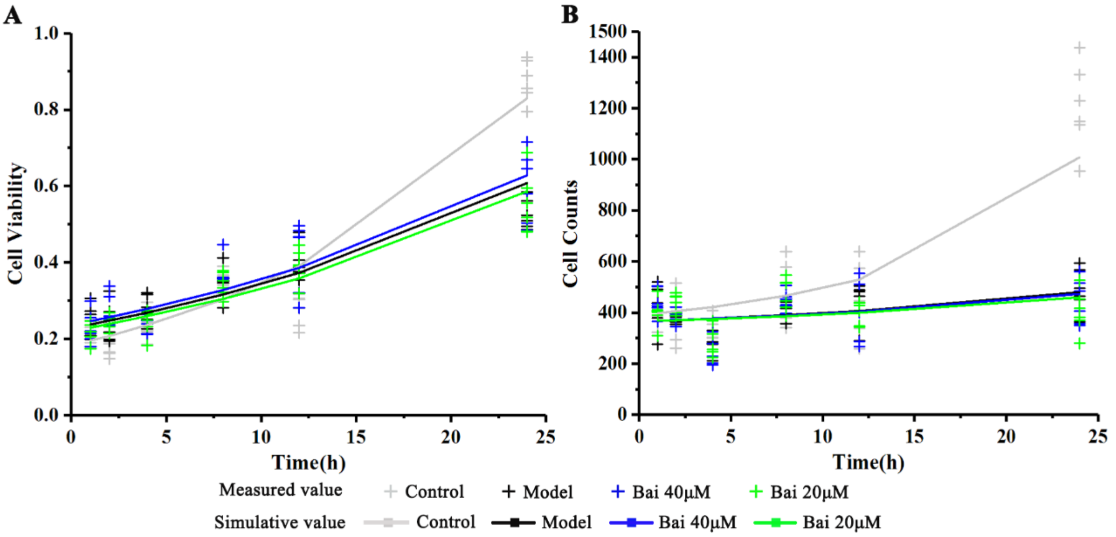

FIGURE S1 | Anti-inflammatory effect of baicalein on the experiment measured and model-simulated cell viability and cell counts in LPS induced RAW264.7 macrophages.

For this model, the goodness-of-fit plots of cell viability and cell counts are shown in **Figure S2**. In the model, the dots of observed data (OBS) versus either the population predicted value (PRED) or individual predicted value (IPRE) distributed around x=y, and the trend lines closely coincide with x=y. The conditional weighted residuals (CWRES) were randomly and homogenously distributed around x axis, and the trend lines closely coincide with x-axis. The observed values of the cell viability were well correlated with population and individual predicted values. The model parameters were also estimated with good precision (Table S1). The coefficient of variation (CV%) of each pharmacokinetic parameter estimate was ≤ 45%, which means the model adequately described the predictions of the cell viability and provided credible parameter estimates. (-2xlog-likelihood=2408.05; Akaike Information Criteria (AIC)= 2450.05, and Bayesian Information Criteria (BIC)= 2461.91).

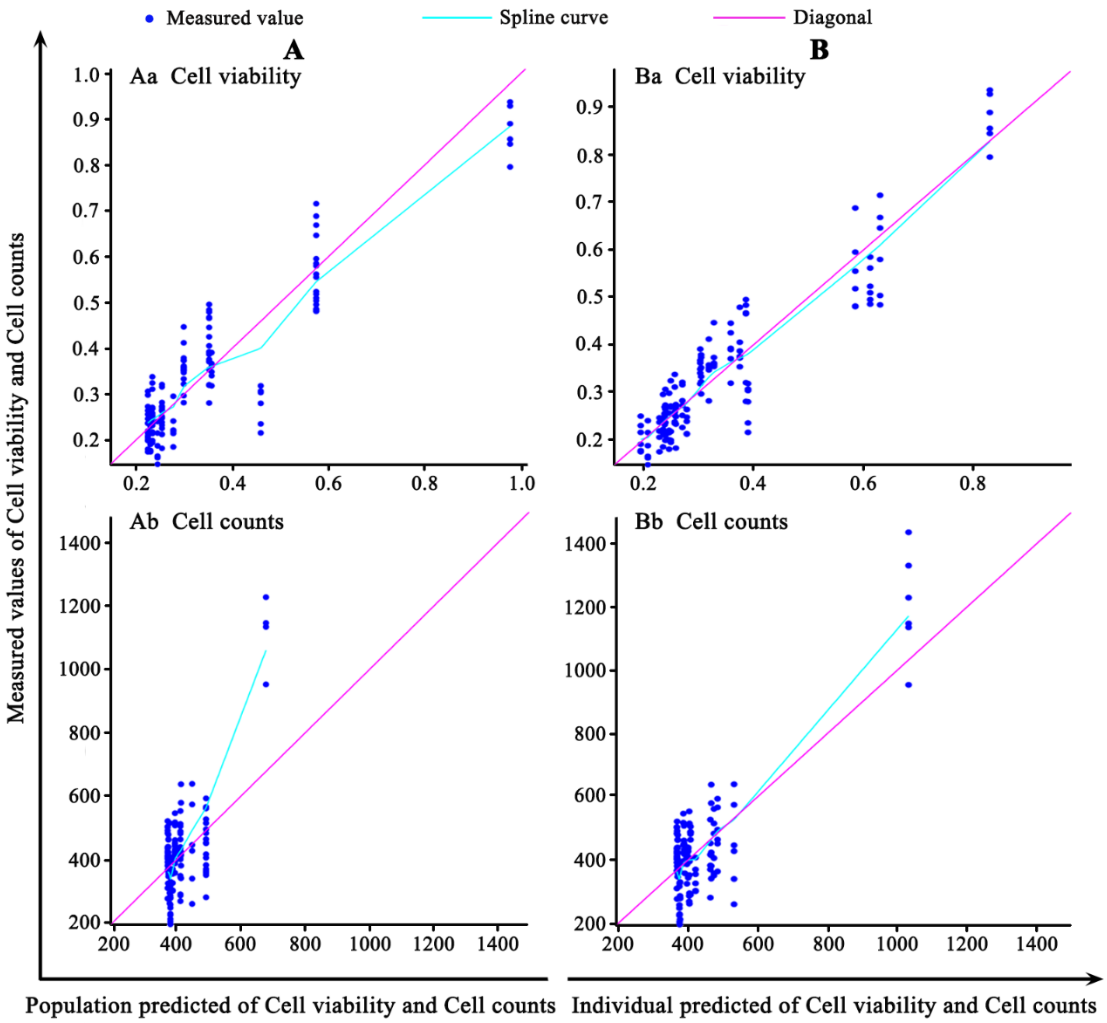

FIGURE S2 | The goodness-of-fit plots of the pharmacodynamic model: Relationship between observed and predicted population values of cell viability (Aa) and cell counts (Ab); Relationship between observed and predicted individual values of cell viability (Ba) and cell counts (Bb).

TABLE S1 | Estimates of parameters of cell viability and cell counts for Pharmacodynamics models

Parameter	Definition	Estimate	CV%
α	Exponential growth rate constant of the cell viability	0.216	5.48
β	Amplificatory coefficient of the exponential equation of cell viability	0.0629	5.13
γ	Constant of inhibitory effect of LPS on the cell	0.648	6.83
d	Exponential growth rate constant of the cell counts	310	5.12
f	Amplificatory coefficient of the exponential equation of cell counts	0.8	25.52

To further verify the results of our study, we have compared two different sequence of LPS stimulation. In this study, cells were firstly stimulated with LPS for 0.5h or 4h and then incubated with baicalein for another 6 and 8h to verify the important role of TNF-α. IL-6 and NO concentration

were quantified and shown in **Figure S3** and **Figure S4**, respectively. The results showed that the inhibitory effect of baicalein with an early(0.5h) LPS stimulation was actually stronger than that with a delay (4h) LPS stimulation. We speculated that the early LPS stimulation could induce the rapid rises of TNF-α level, which could regulate, at least in part, the next step of inflammation. But the delay LPS stimulation could not induce the next step of IL-6 and NO, because the increase of TNF-α with the peak after1h LPS stimulation, has gone.

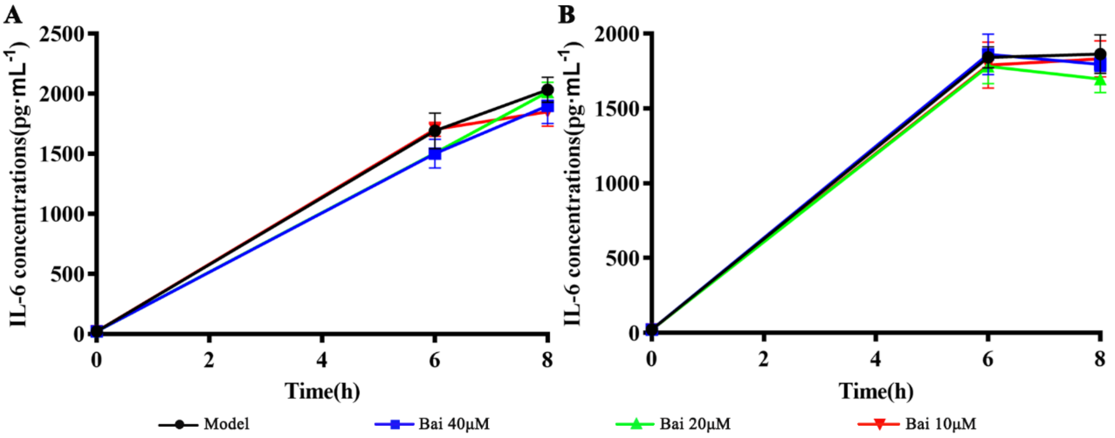

FIGURE S3 | Effects of baicalein on IL-6 concentrations after an early and delay stimulation of LPS. RAW264.7 macrophages cells were stimulated with LPS for 0.5h (A) and 4h(B), and then incubated with 40, 20, 10 and 0 (control) μM baicalein for another 6 and 8h. IL-6 concentration were quantified by ELISA. For experimental data points, n = 3.

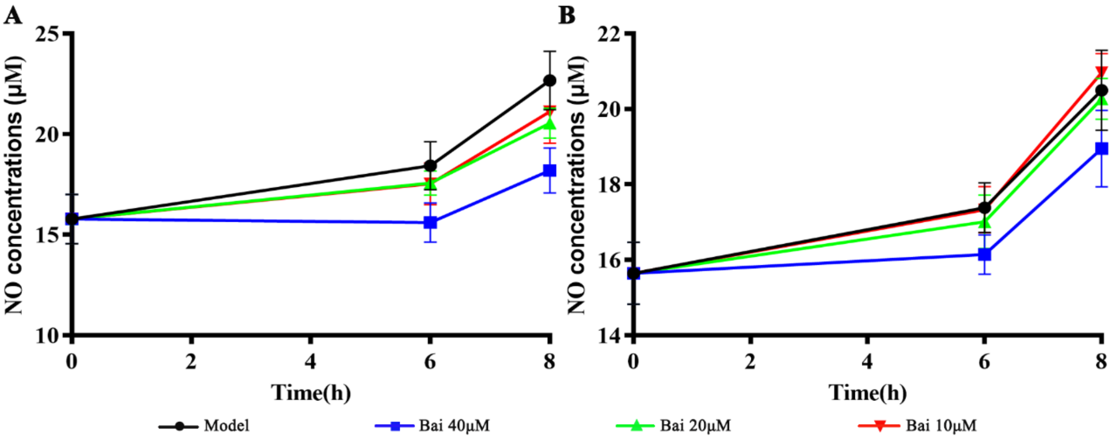

FIGURE S4 | Effects of baicalein on NO concentrations after an early and delay stimulation of LPS. Effects of baicalein on NO concentrations after a delay stimulation of LPS. RAW264.7 macrophages cells were stimulated with LPS for 0.5h (A) and 4h(B), and then incubated with 40, 20, 10 and 0 (control) μM baicalein for another 6 and 8h. NO concentration were quantified by Griess. For experimental data points, n = 3.

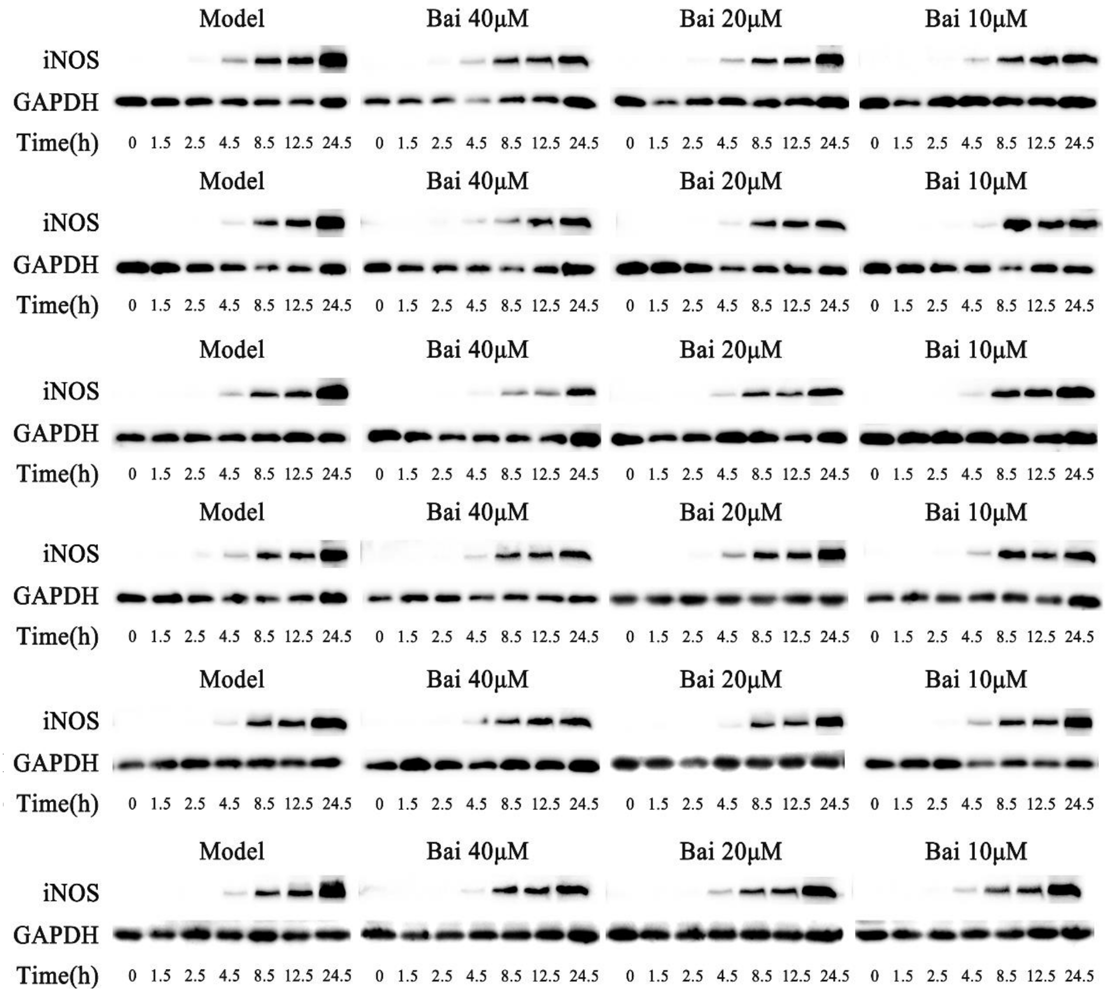

FIGURE S5 | The six times expression of iNOS.

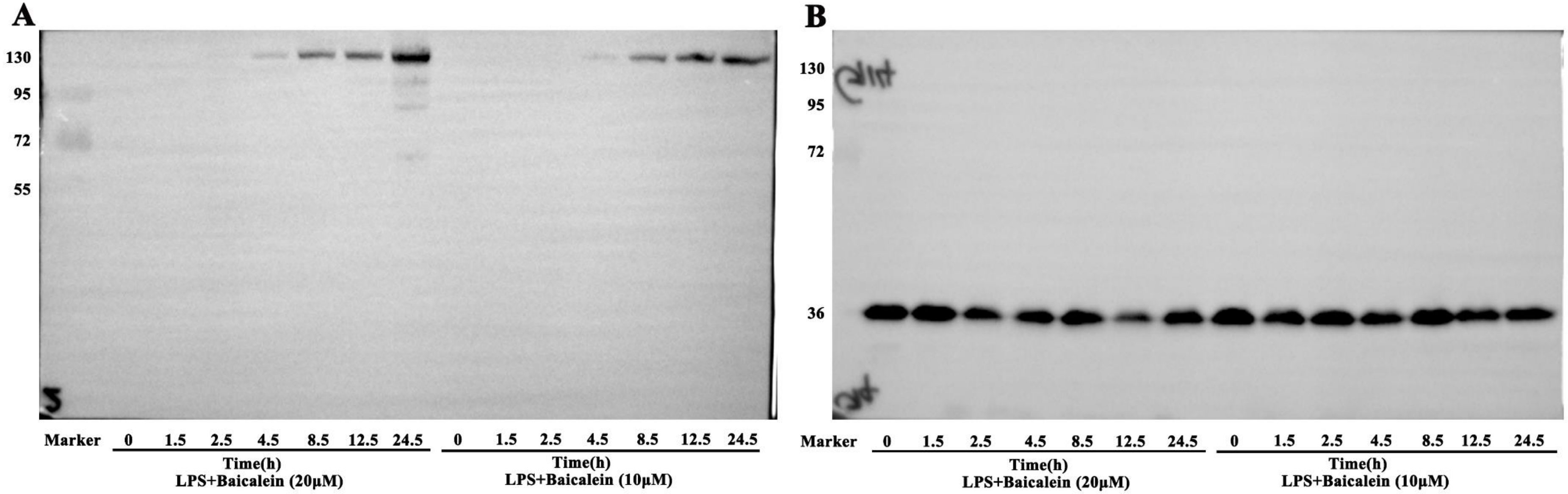

FIGURE S6 | The uncropped PVDF membrane of the expression of iNOS(A) and GAPDH(B).
